# Supplementary material for: Heterogeneity and Differentiation Trajectories of Infiltrating CD8+ T Cells in Lung Adenocarcinoma
Source: Cancers (Basel). 2022 Oct 22;14(21):5183. doi: 10.3390/cancers14215183 (PMC9658355; doi:10.3390/cancers14215183)
Supplement: Supplementary file 1 [file cancers-14-05183-s001.zip › Supplementary figure legends.pdf]

Figure S1 Processing of single-cell data of GSE131907. (A) The total number of genes in the three groups of lung cancer samples. (B) Number of gene sequences in three groups of lung cancer samples. (C) The proportion of mitochondrial genes in total genes in the three groups. (D) The correlation coefficient between the number of Count and the proportion of mitochondrial genes was 0.03, indicating that mitochondrial genes have been deleted. (E) The correlation coefficient between the number of Count and the number of genes obtained was 0.89, indicating that the sequencing depth was reliable. (F) PCA analysis of 2000 genes with large variation. All 20 PCs were significantly different and could be used for subsequent analysis. (G) Cluster map of all cells. (H) Distribution of CD8A and CD8B genes in the cluster map.

Figure S2 The distribution of marker gene with pseudo time. (A-D) The distribution of naive-like markers CCR7, LEF1, TCF7 and SELL with pseudo time, and their expression levels gradually decreased with the progress of cell differentiation. (E-H) Distribution of cytotoxic markers PRF1, GZMA, GZMK and NKG7 with pseudo time, and their expression levels fluctuated but persisted with the progress of cell differentiation. (I-L) The distribution of exhausted markers CTLA4, PDCD1, LAG3 and HAVCR2 with pseudo time, and their expression levels gradually increased with the process of cell differentiation.

Figure S3 The expression heatmap of markers of short-lived effector cells (SLECs), memory precursor effector cells (MPECs), progenitor exhausted CD8+ T cells and terminally exhausted CD8+ T cells.

Figure S4 Cell-cell interaction network analysis. (A) Interactions among ten CD8+ T cell subsets. (B) Heatmap of ligand-receptor pairs among ten CD8+ T cell subsets. (C) Histogram of the number of ligand-receptor pairs. (D) Heatmap of transcription factor expression in ten CD8+ T cell subsets.

Figure S5 Functional enrichment and metabolic pathway activity analysis of ten CD8+ T cell subsets. (A) GO analysis of ten CD8+ T cell subsets. (B) KEGG analysis of ten CD8+ T cell subsets. (C) Metabolic pathways with significant differences in activity among ten CD8+ T cell subsets. (D) Significantly different Hallmark pathway and immune checkpoint pathway among ten CD8+ T cell subsets.

Figure S6 Prognostic analysis of 6 subsets and correlation analysis of ETL subset with clinical data. (A) The effect of CTL1 subset proportion on the OS of LUAD patients, *P*=0.78. (B) The effect of CTL2 subset proportion on the OS of LUAD patients, *P*=0.89. (C) The effect of CTL3 subset proportion on the OS of LUAD patients, *P*=0.39. (D) The effect of CTL4 subset proportion on the OS of LUAD patients, *P*=0.38. (E) The effect of CTL6 subset proportion on the OS of LUAD patients, *P*=0.11. (F) The effect of NTL subset proportion on the OS of LUAD patients, P=0.82. (G) Distribution of ETL subset in different T stages, M stages and AJCC stages of LUAD patients.

Figure S7 The relative expression of four hub genes in GSE43458 dataset. (A) The relative expression levels of IL7R in the three groups of samples. (B) The relative expression levels of AGER in the three groups of samples. (C) The relative expression levels of GAPDH in the three groups of samples. (D) The relative expression levels of

CD69 in the three groups of samples.

Figure S8 Associated genes for GSEA analysis of 4 hub genes. (A) Associated genes of pathways enriched when AGER is highly expressed. (B) Associated genes of pathways enriched when CD69 is highly expressed. (C) Associated genes of pathways enriched when GAPDH is highly expressed. (D) Associated genes of pathways enriched when IL7R is highly expressed.
