# Supplementary material for: Heterogeneity and Differentiation Trajectories of Infiltrating CD8+ T Cells in Lung Adenocarcinoma
Source: Cancers (Basel). 2022 Oct 22;14(21):5183. doi: 10.3390/cancers14215183 (PMC9658355; doi:10.3390/cancers14215183)
Supplement: Supplementary file 1 [file cancers-14-05183-s001.zip › Table S4.pdf]

Table S4. Clinical data of 24 patients involved in this manuscript

Patients[1]	Gender	Age	Time of first diagnosis[2]	Pathology results[3]	Immunohistochemical results[4]	TNM[5]	Chemotherapy regimens[6]
#1	Male	63	30 Jul 2021	AIS	TTF-1(+)、NapsinA(+)、CK7(+)、CK5/6(-)、P63(-)、 Ki-67(+，5%)	pT1bN0M0 IA	-
#2	Male	42	28 Jul 2021	AIS	TTF-1(+)、NapsinA(+)、CK7(+)、EGFR(+)、P40(-)、 Ki-67(+，1%)	pT1aN0M0 IA	-
#3	Female	71	28 Jul 2021	IAC	TTF-1(+)、NapsinA(+)、CK7(+)、EGFR(+)、P40(-)、 Ki-67(+，5%)	pT1bN0M0 IA	-
#4	Male	63	23 Jul 2021	IAC	TTF-1(-)、NapsinA(-)、CK7(+)、EGFR(1+)、Ki-67(+，40%)	pT1aN0M0 IA	-
#5	Male	65	20 Jul 2021	IAC	TTF-1(+)、CK7(+)、EGFR(3+)、Ki-67(+，25%)	pT1bN0M0 IA	-
#6	Male	59	1 Jul 2021	IAC	CK7(+)、 EGFR(2+)、Ki-67(+，30%)	pT1aN0M0 IA	-
#7	Female	67	25 Jun 2021	IAC	TTF-1(+)、NapsinA(+)、CK7(+)、EGFR(2+)、Ki-67(+，10%)	pT1bN0M0 IA	-
#8	Female	56	22 Jun 2021	IAC	TTF-1(+)、NapsinA(+)、CK7(+)、SATB2(-)、Ki-67(+，10%)	pT1bN0M0 IA	-
#9	Female	78	22 Jun 2021	IAC	TTF-1(+)、NapsinA(+)、CK7(+)、SATB2(-)、Ki-67(+，15%)	pT2aN0M0 IB	-
#10	Male	56	18 Jun 2021	IAC	TTF-1(+)、NapsinA(+)、CK7(+)、EMA(+)、EGFR(+)、 Ki-67(+，10%)	pT2aN1M0 IIB	AP*4
#11	Female	53	8 Jun 2021	IAC	TTF-1(+)、CK7(+)、EGFR(3+)、Ki-67(20%+)	pT1bN0M0 IA	-
#12	Female	53	7 Jun 2021	AIS	TTF-1(+)、 NapsinA(+)、 P63(-)、 Ki-67(+，5%)	pT1aN0M0 IA	-

#13	Male	72	1 Jun 2021	IAC	TTF-1(+)、NapsinA(+)、CK7(+)、EGFR(3+)、Ki-67(80%)	pT1bN1M0 IIB	AP*4
#14	Male	56	26 May 2021	IAC	TTF-1(+)、NapsinA(+)、 CK7(+)、EGFR(+)、Ki-67(+，30%)	pT1bN0M0 IA	-
#15	Female	64	20 May 2021	IAC	TTF-1(+)、NapsinA(+)、CEA(+)、CK7(+)、EGFR(+)、Ki-67(+，15%)	pT1bN0M0 IA	-
#16	Female	66	17 May 2021	IAC	TTF-1(+)、NapsinA(+)、CK7(+)、EGFR(+)、Ki-67(+，5%)	pT1bN0M0 IA	-
#17	Female	68	17 May 2021	IAC	TTF-1(+)、NapsinA(+)、CK7(+)、EGFR(2+)、CK5/6(-)、P40(-)、Ki-67(+，20%)	pT1bN0M0 IA	-
#18	Male	56	8 May 2021	IAC	TTF-1(+)、NapsinA(+)、CK7(+)、EGFR(2+)、Ki-67(10%)	pT1bN0M0 IA	-
#19	Female	47	30 Apr 2021	IAC	TTF-1(+)、NapsinA(+)、CK7(+)、EGFR(2+)、Ki-67(+，30%)	pT2aN0M0 IB	TKI
#20	Male	64	28 Apr 2021	IAC	TTF-1(+)、NapsinA(+)、CK7(+)、EGFR(+)、Ki-67(+，30%)	pT2aN0M0 IB	AP*2
#21	Male	67	28 Apr 2021	IAC	TTF-1(+)、NapsinA(+)、CK7(+)、CEA(+)、Ki-67(+，30%)	pT2bN2M0 IIIA	AP*2+TKI
#22	Female	62	26 Apr 2021	IAC	TTF-1(+)、NapsinA(+)、CK7(+)、EGFR(2+)、Ki-67(8%)	pT1bN0M0 IA	-
#23	Male	55	26 Apr 2021	IAC	TTF-1(+)、NapsinA(+)、CK7(+)、EGFR(2+)、Ki-67(5%)	pT1bN0M0 IA	-
#24	Female	58	21 Apr 2021	IAC	TTF-1(+)、NapsinA(+)、CK7(+)、 EGFR(+)、Ki-67(+，5%)	pT2aN2M0 IIIA	-

1 Data were collected from the Case Custodian Department of Qilu Hospital of Shandong University in Qingdao, Tissue samples were fresh and frozen.

Correspondence address: 758 Hefei road, Qingdao, Shandong 200035 P.R. China. Email: qlyyqdxcb@163.com.

2 The patient was first diagnosed with lung adenocarcinoma at Qilu Hospital of Shandong University (Qingdao).

3 Adenocarcinoma in situ, AIS; Invasive adenocarcinoma, IAC.

4 Data were obtained from the Department of Pathology, Qilu Hospital, Shandong University (Qingdao).

5 Staging is based on the AJCC Staging Manual, Edition 8. p stands for pathological stage.

6 Pemetrexed+Cisplatin, AP; Tyrosine kinase inhibitor.
