# Supplementary figures and images for: Heterogeneity and Differentiation Trajectories of Infiltrating CD8+ T Cells in Lung Adenocarcinoma
Source: Cancers (Basel). 2022 Oct 22;14(21):5183. doi: 10.3390/cancers14215183 (PMC9658355; doi:10.3390/cancers14215183)

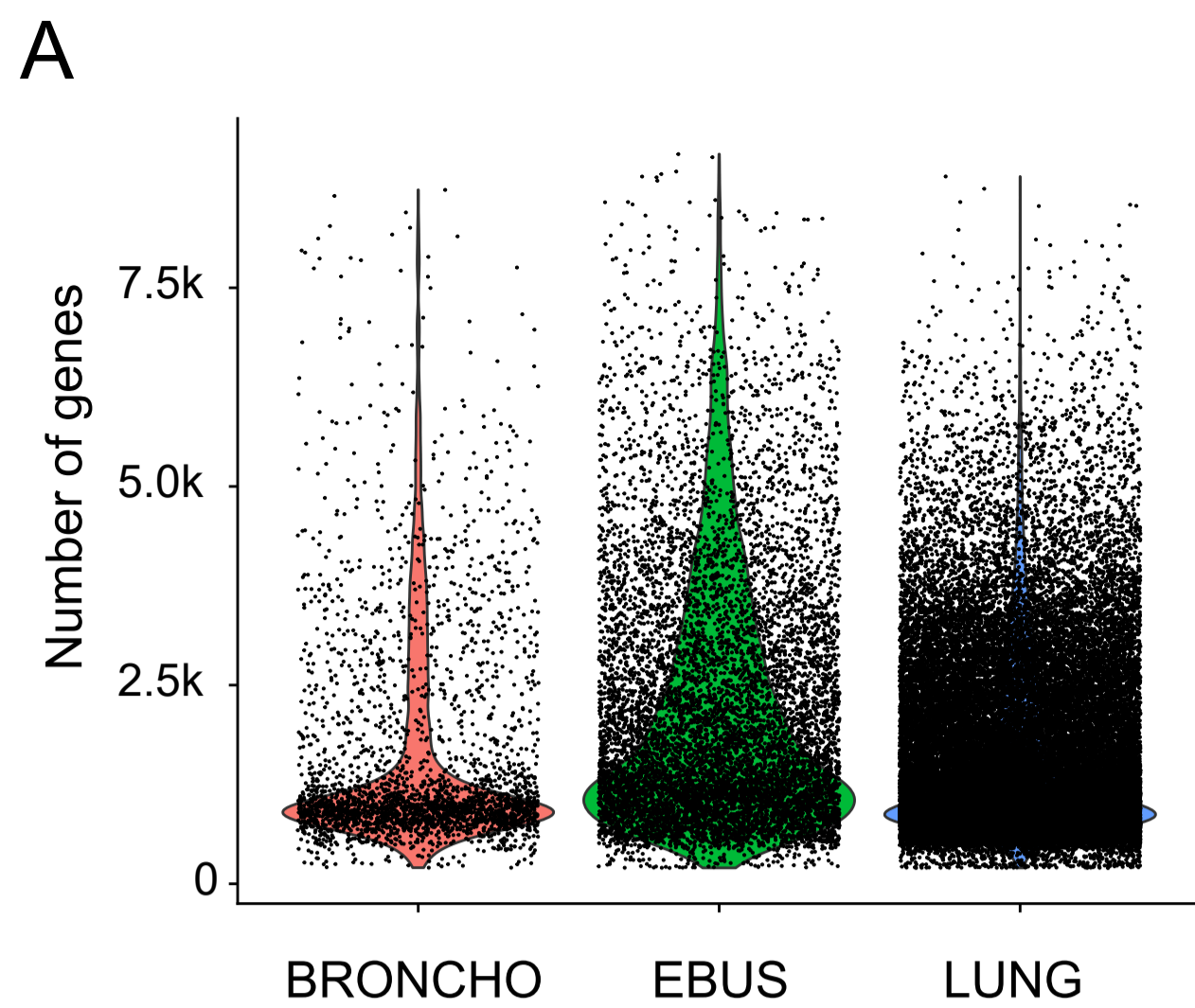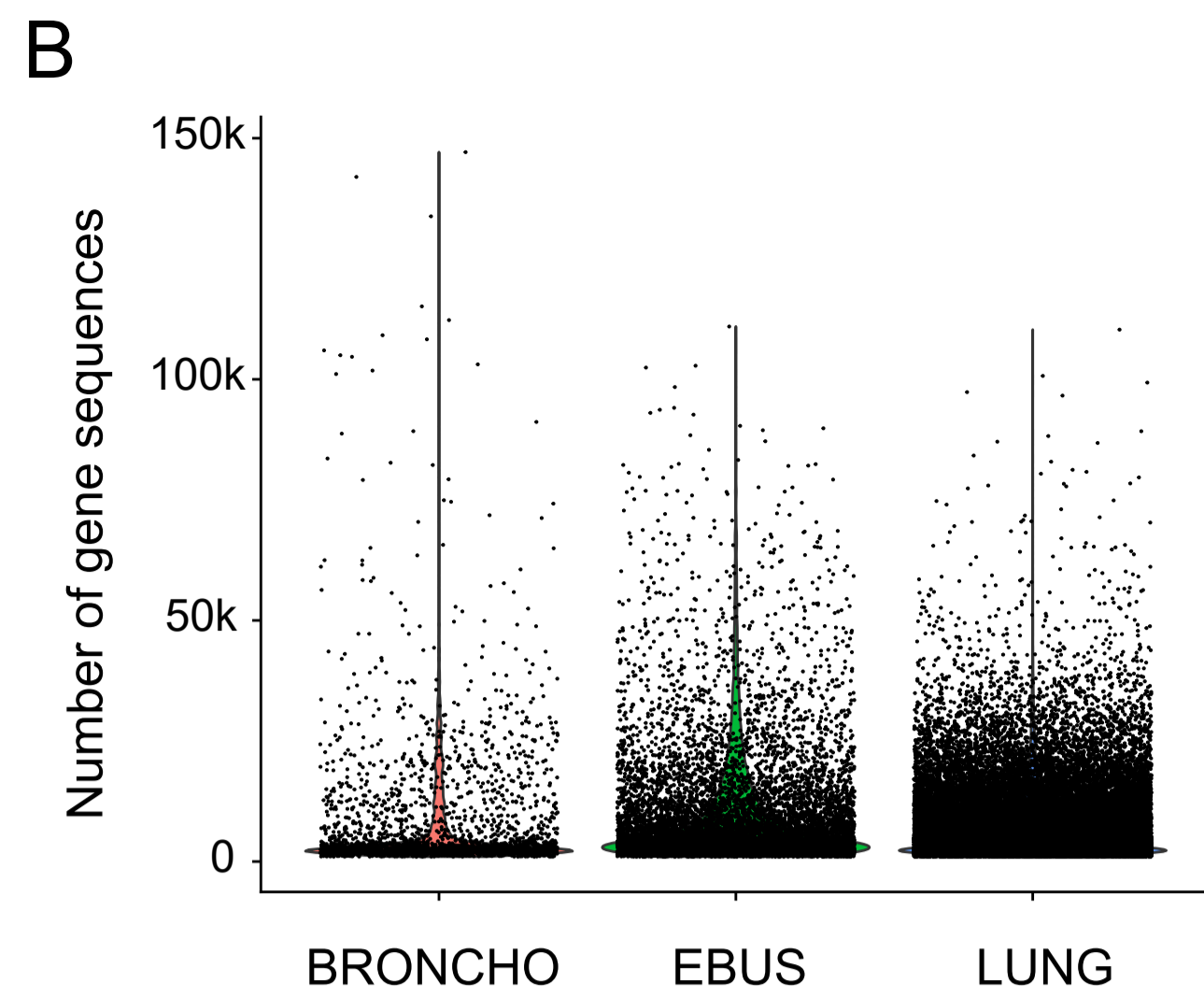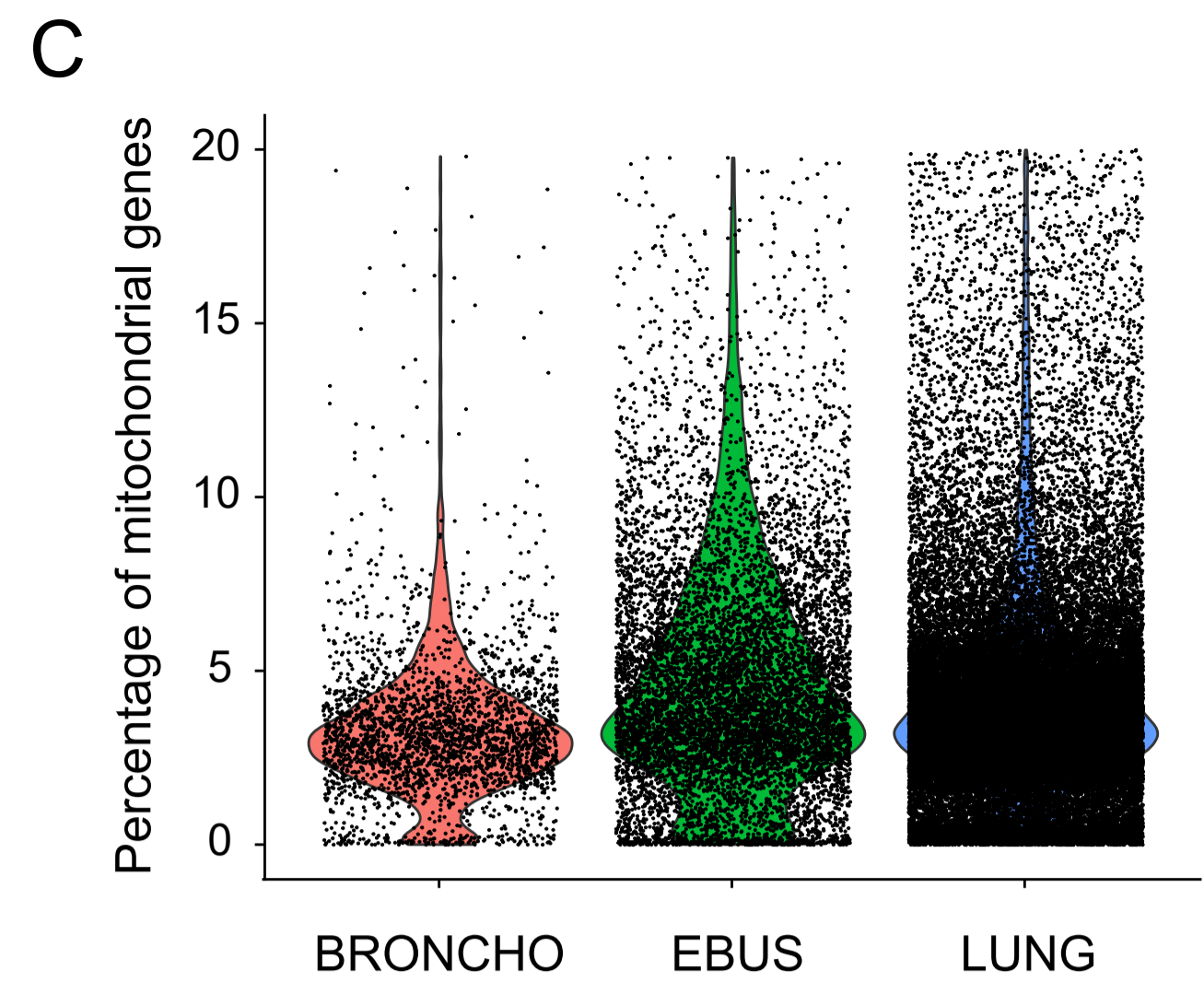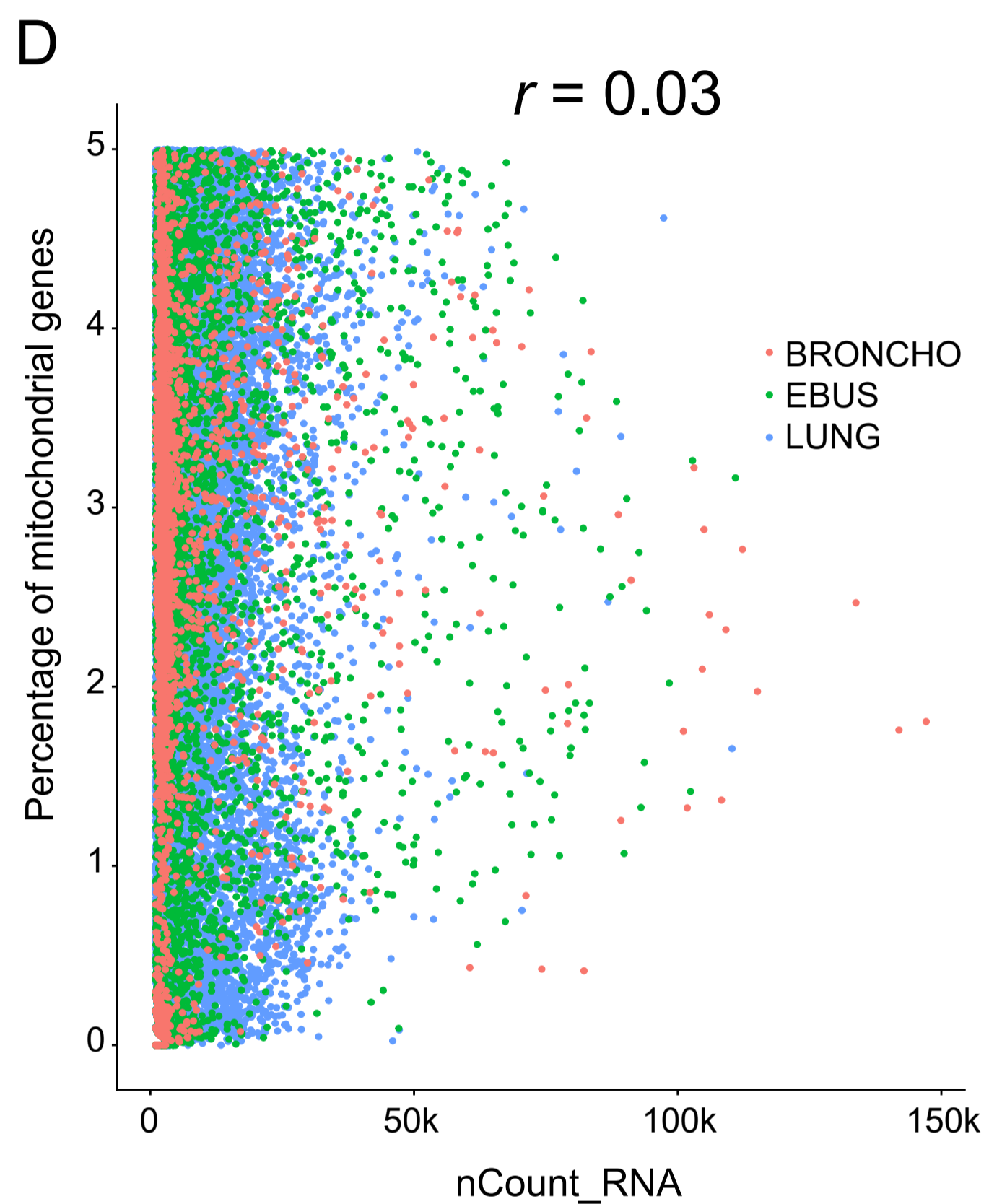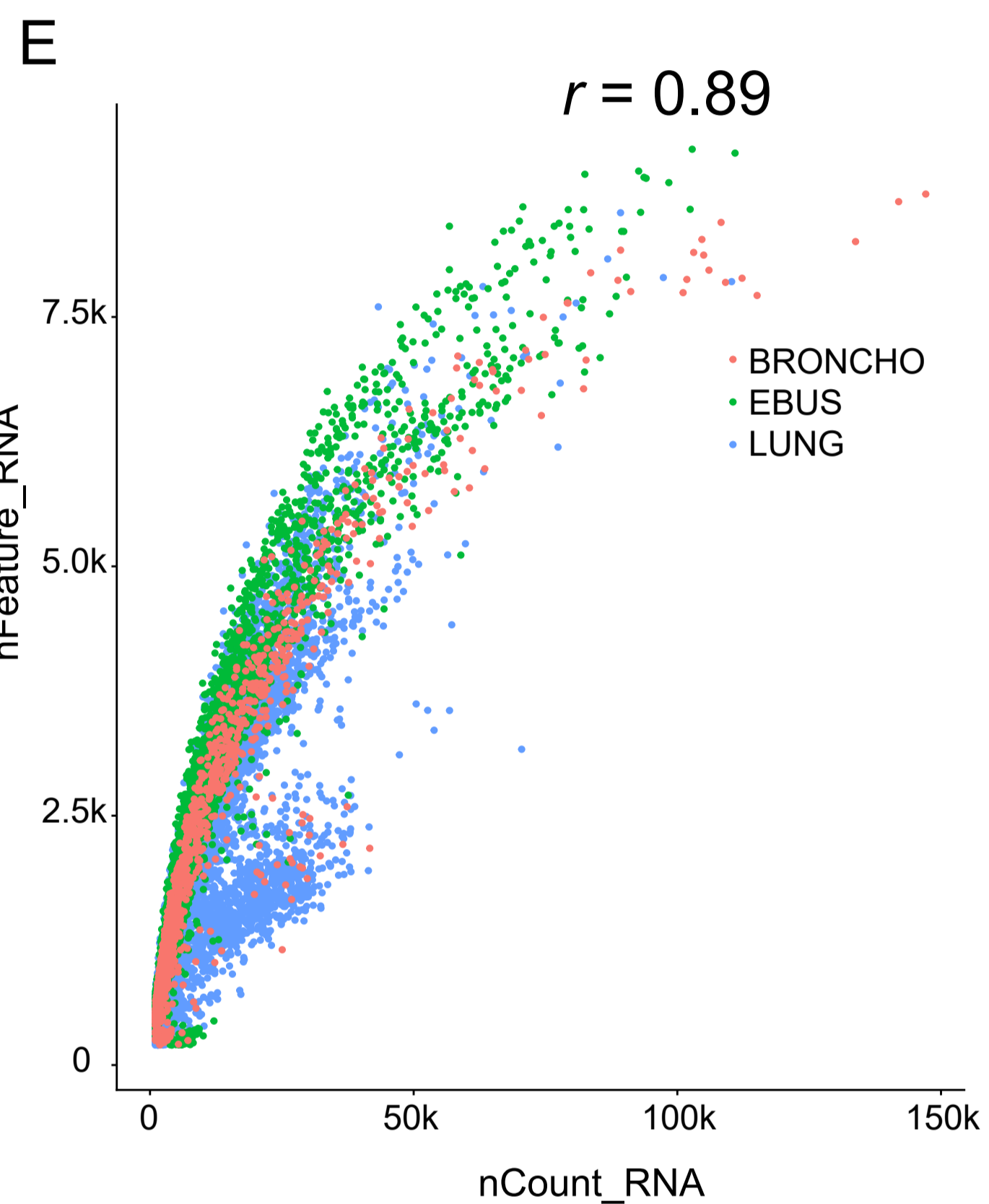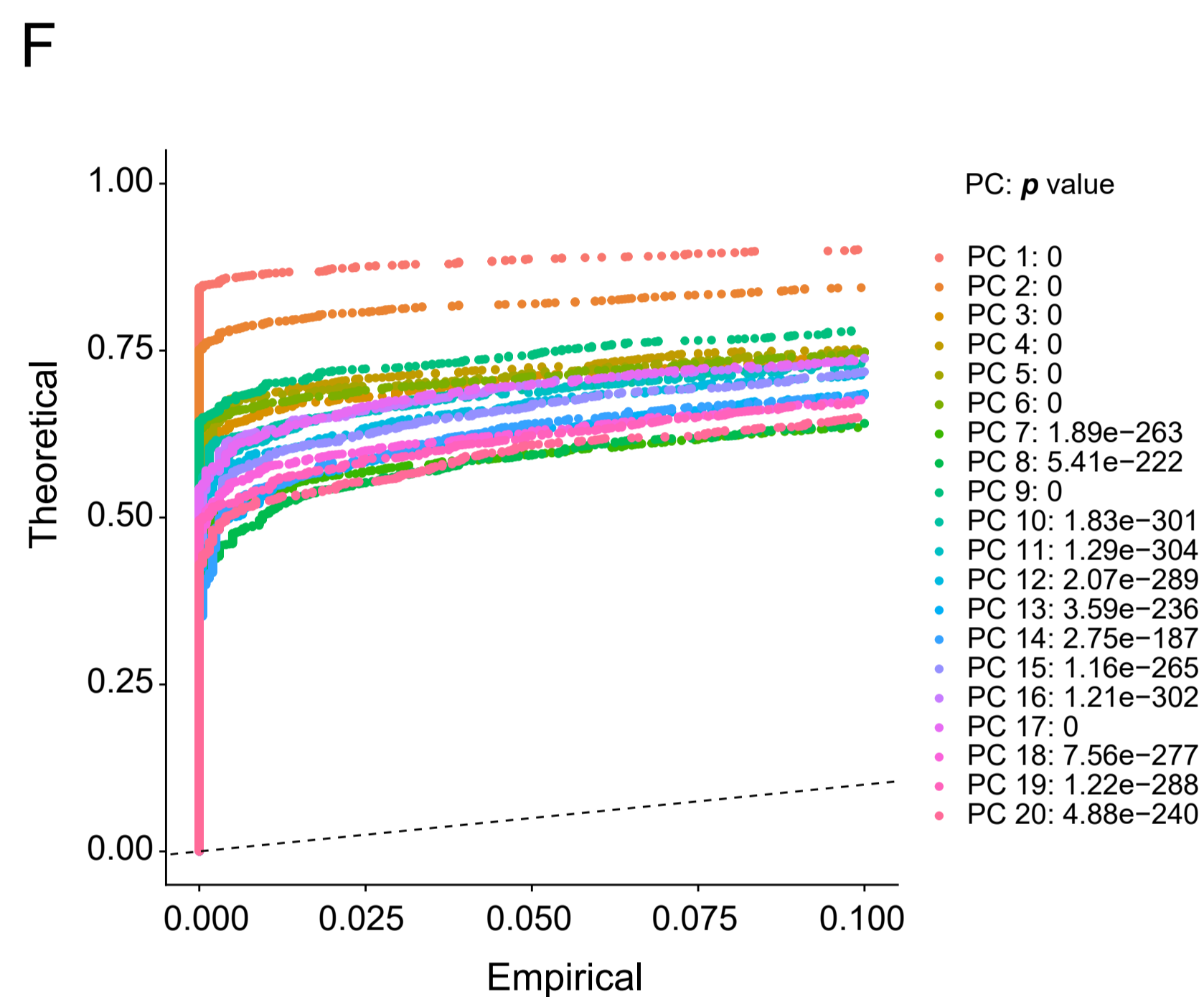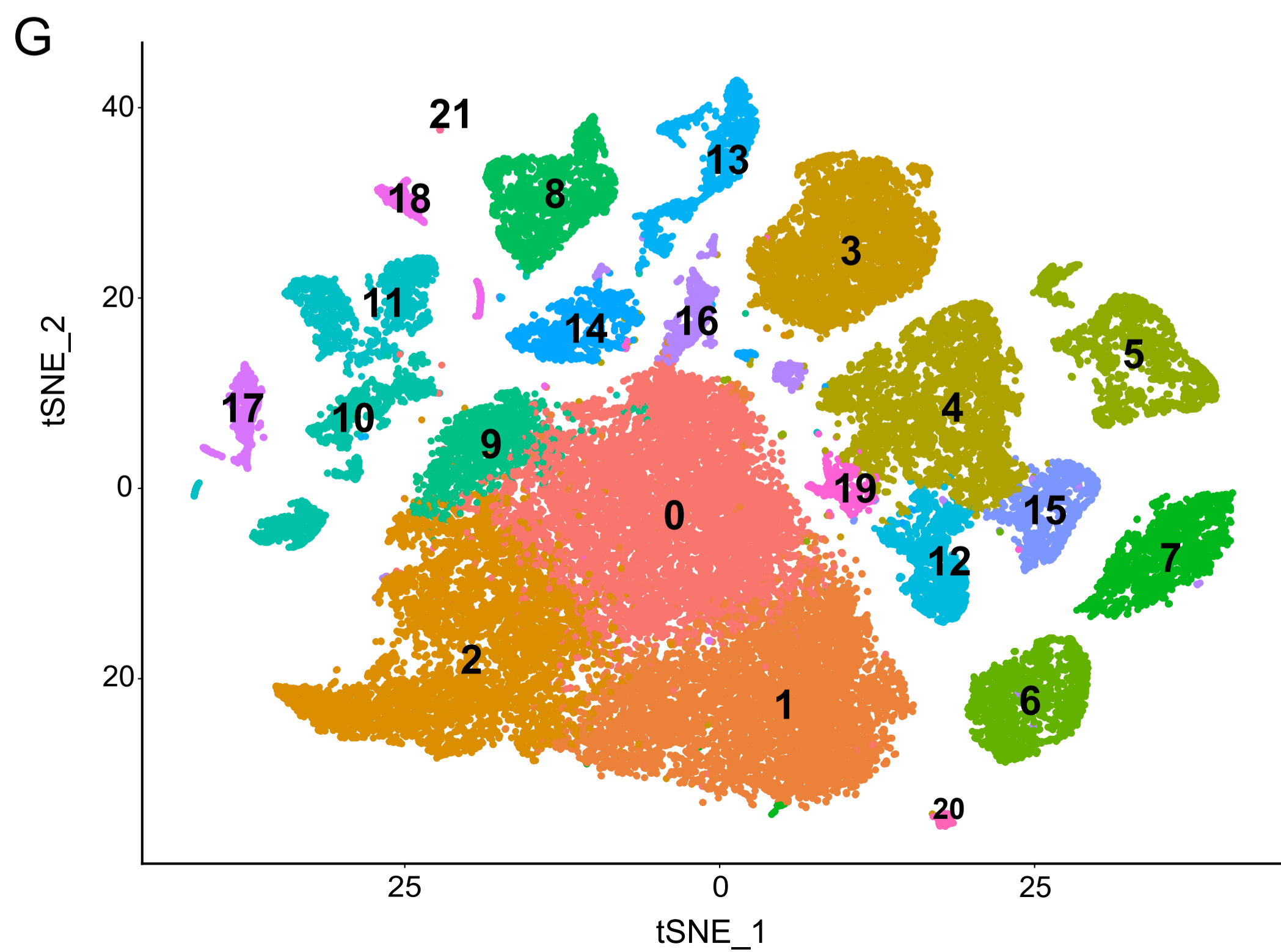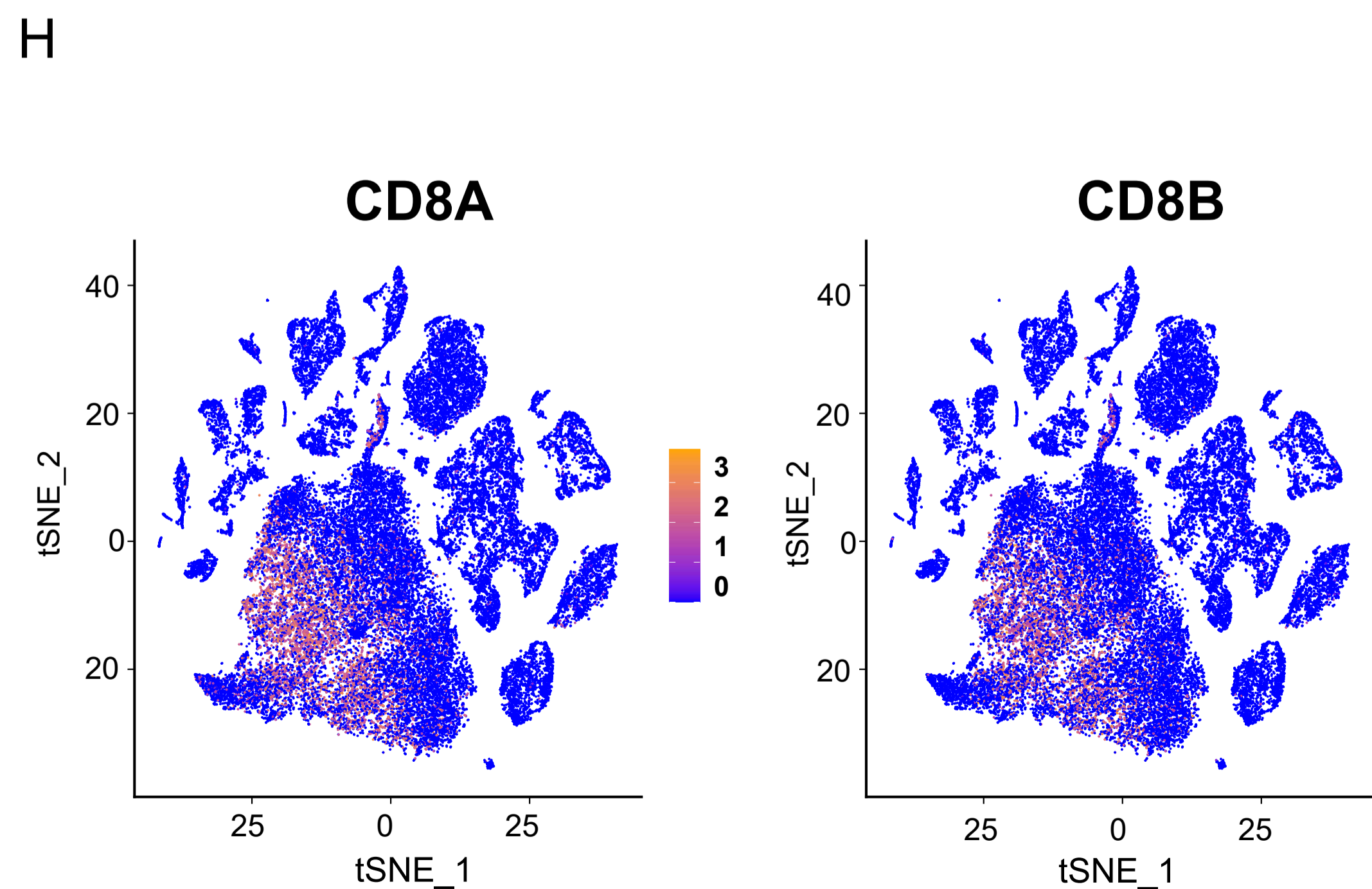

Supplement: Supplementary file 1 [file cancers-14-05183-s001.zip › Figure S1.pdf]

A

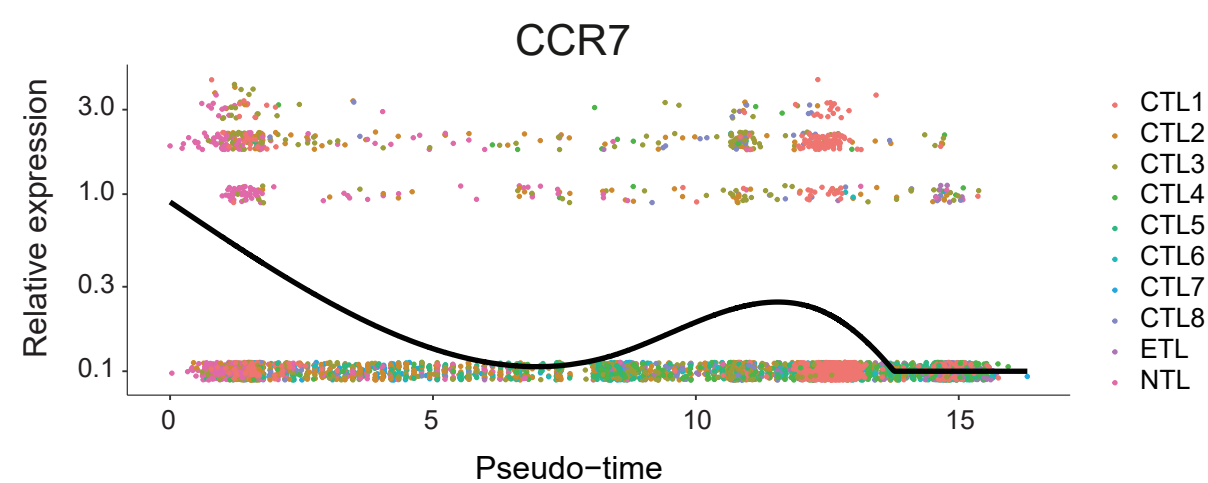

B

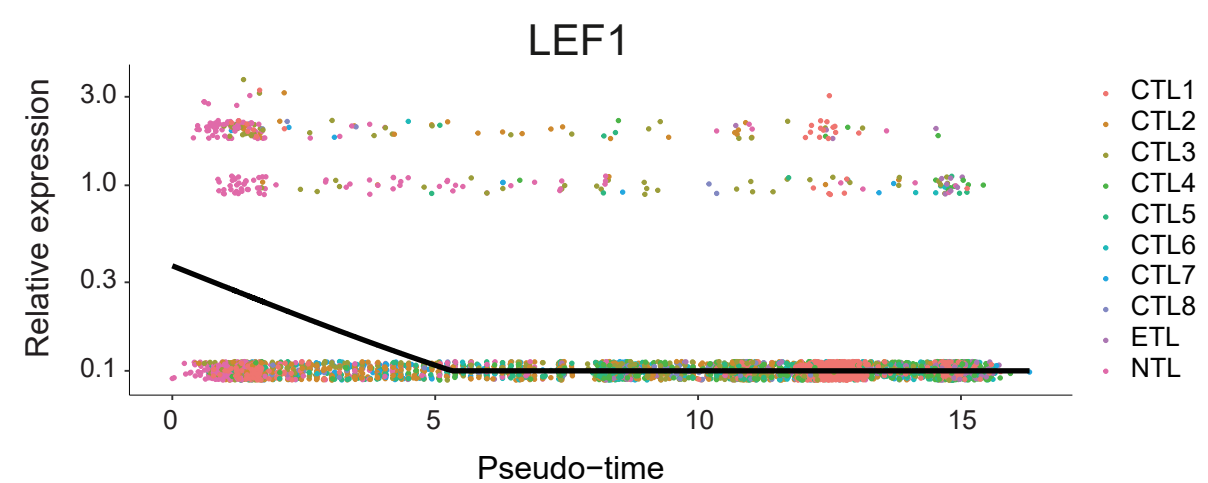

C

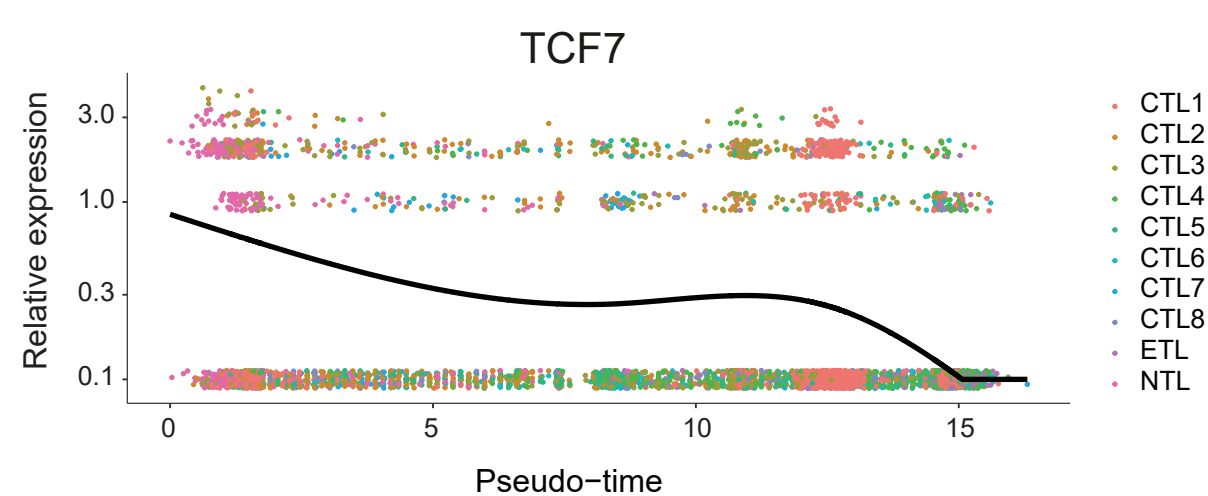

D

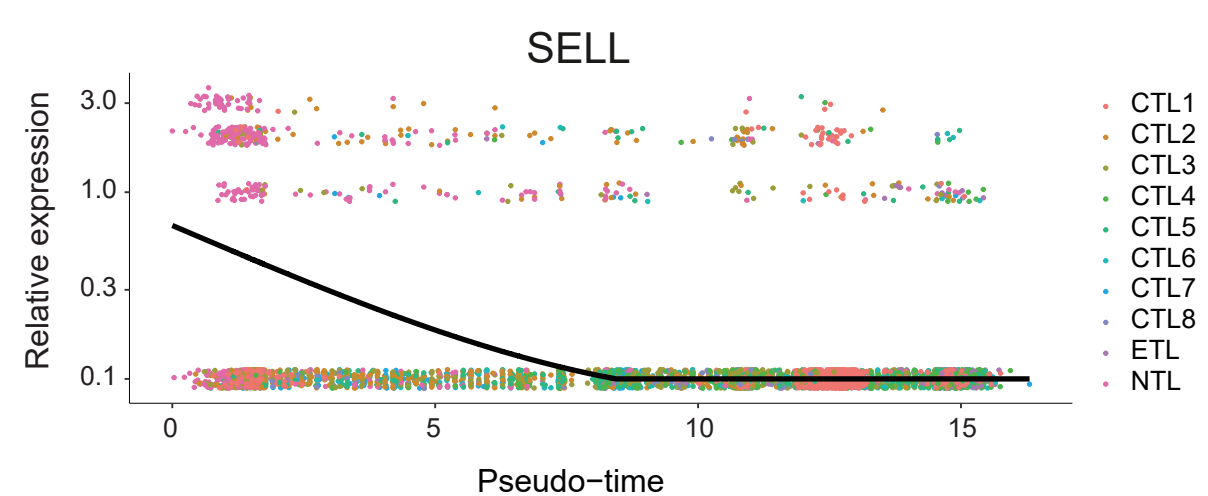

E

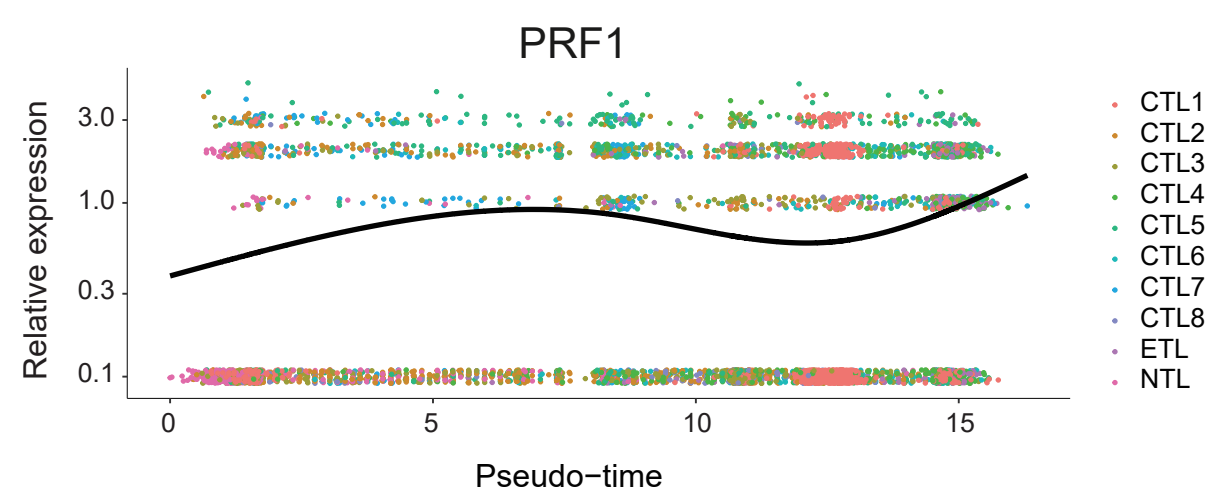

F

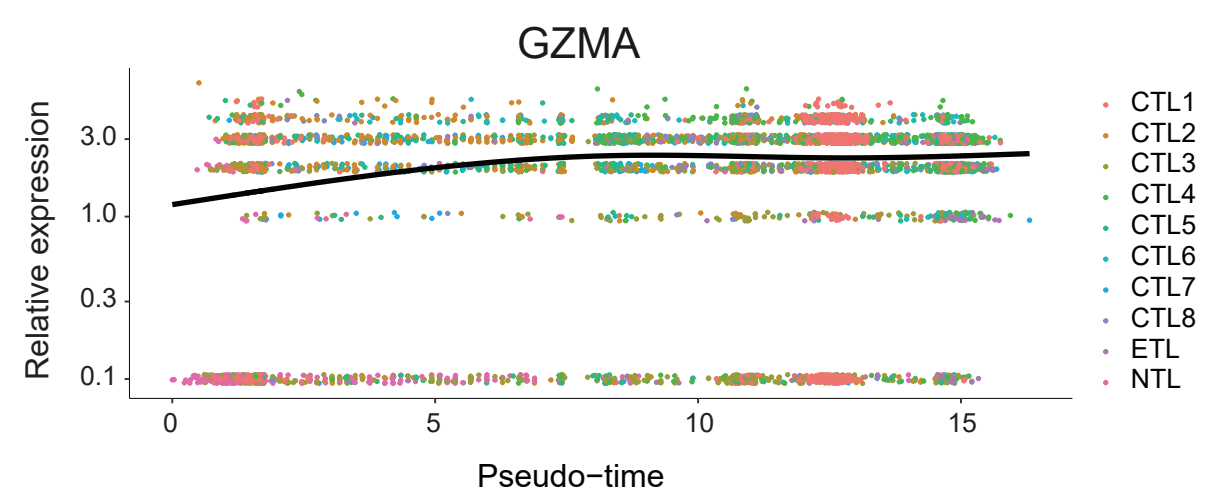

G

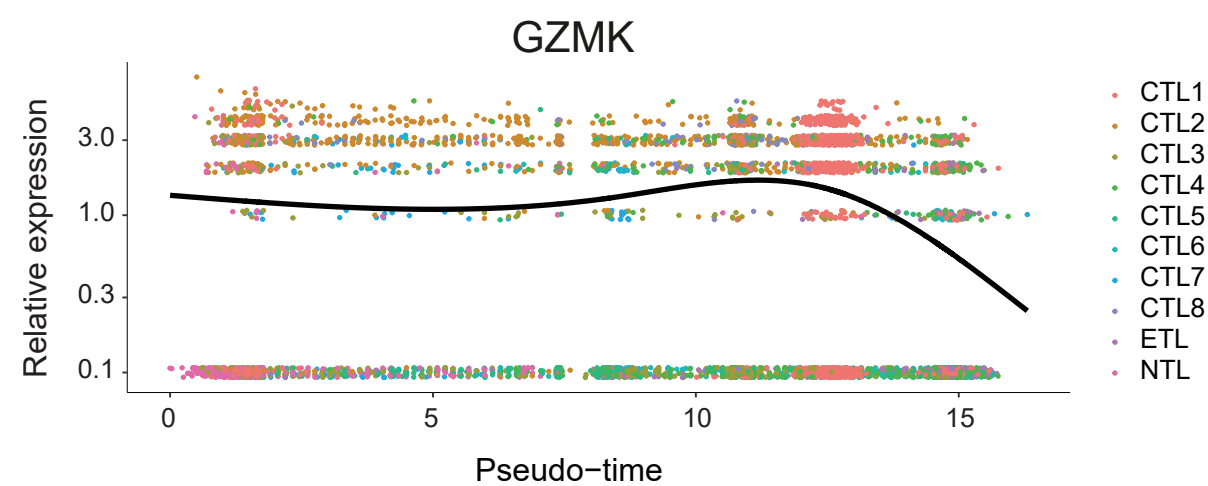

H

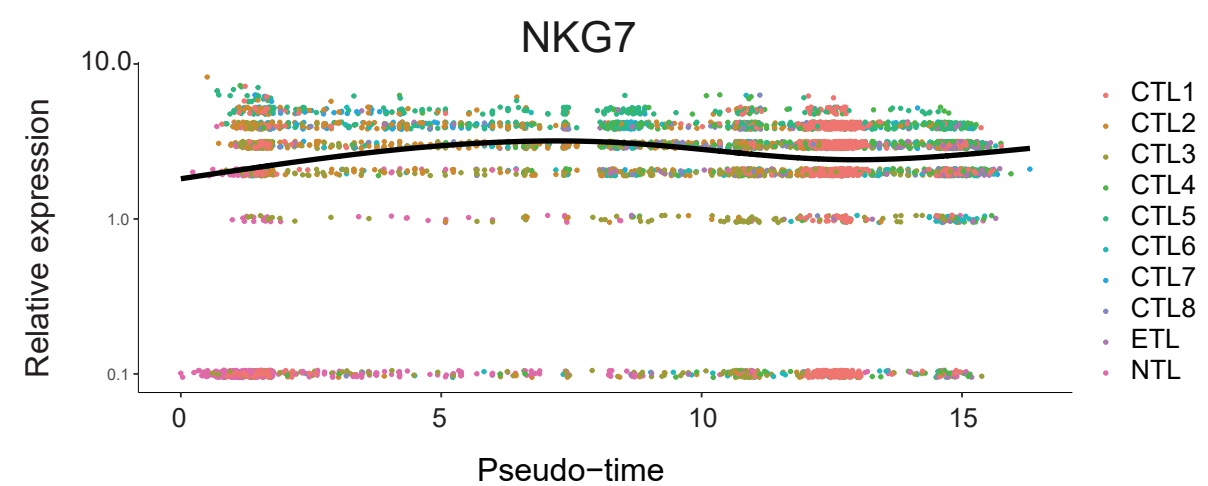

I

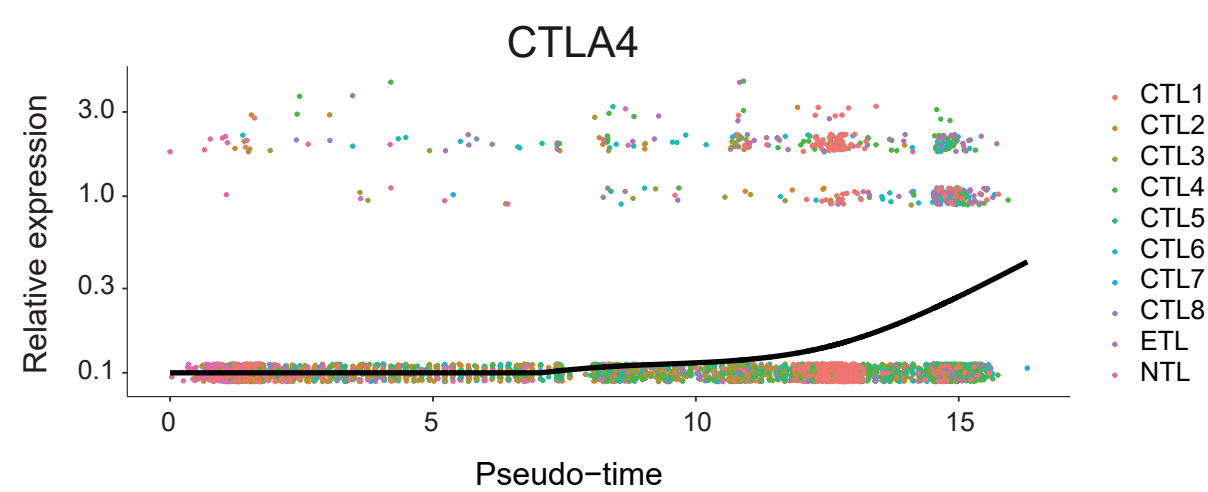

J

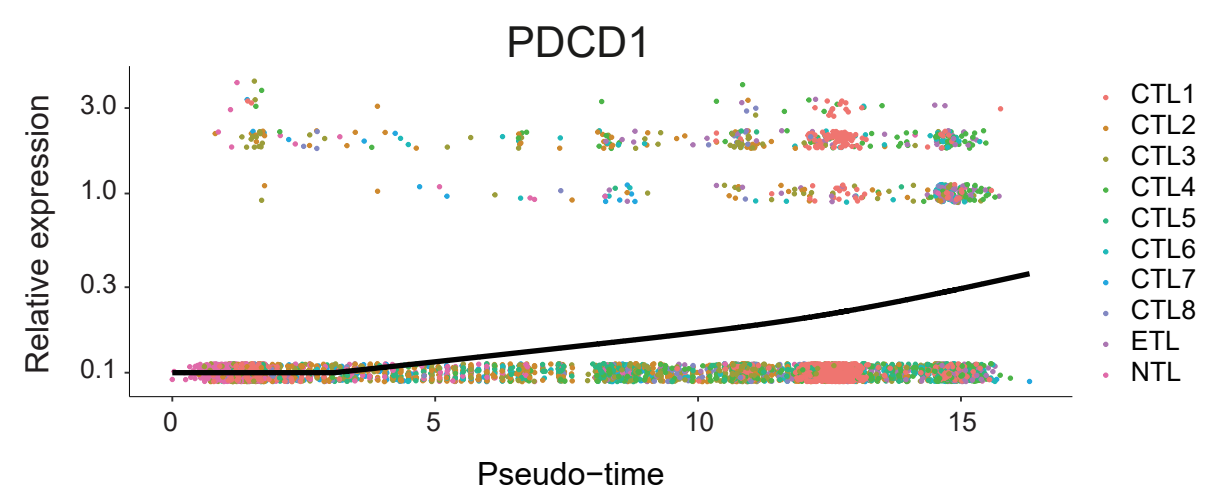

K

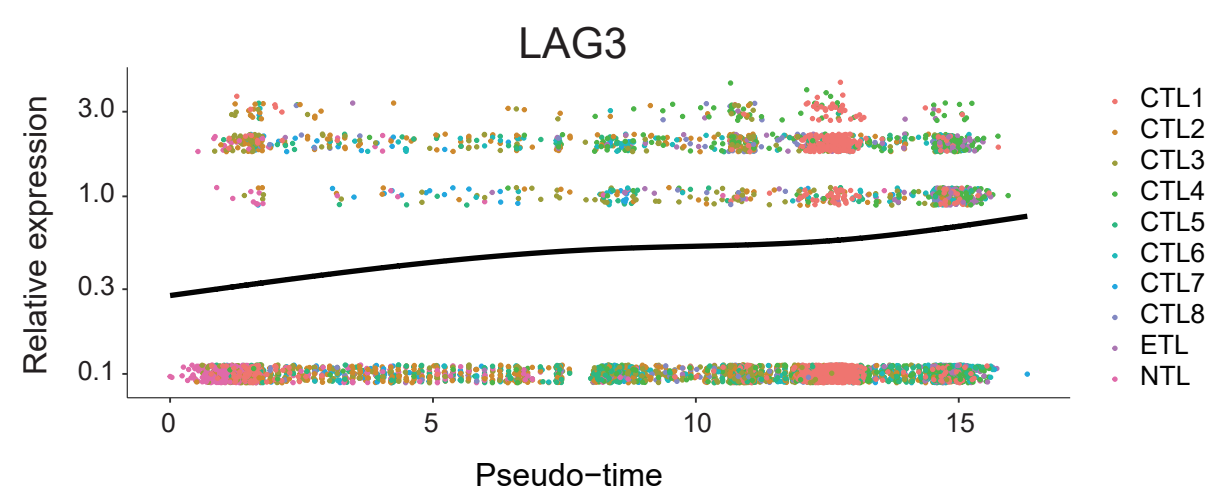

L

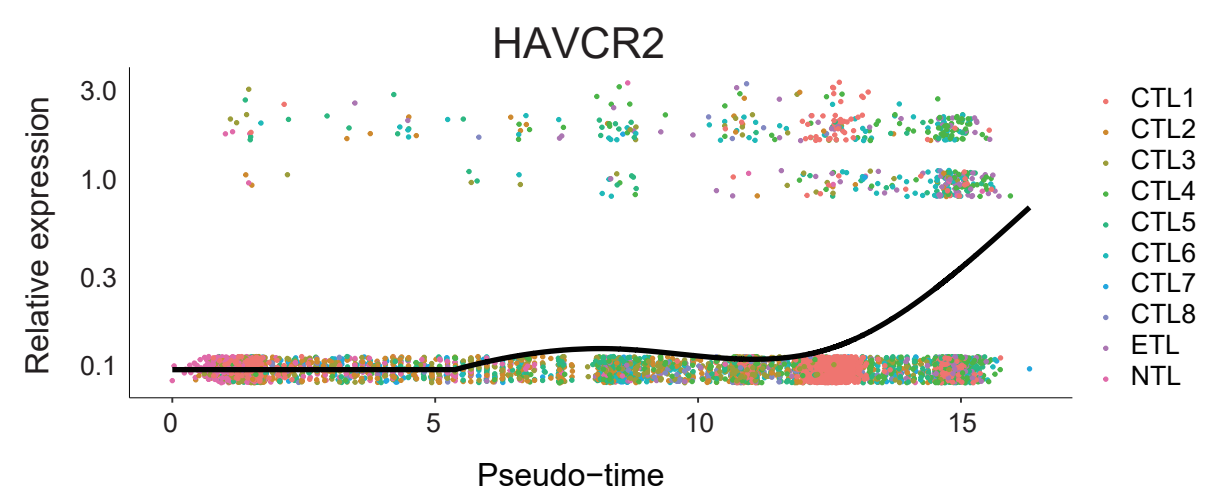

Supplement: Supplementary file 1 [file cancers-14-05183-s001.zip › Figure S2.pdf]

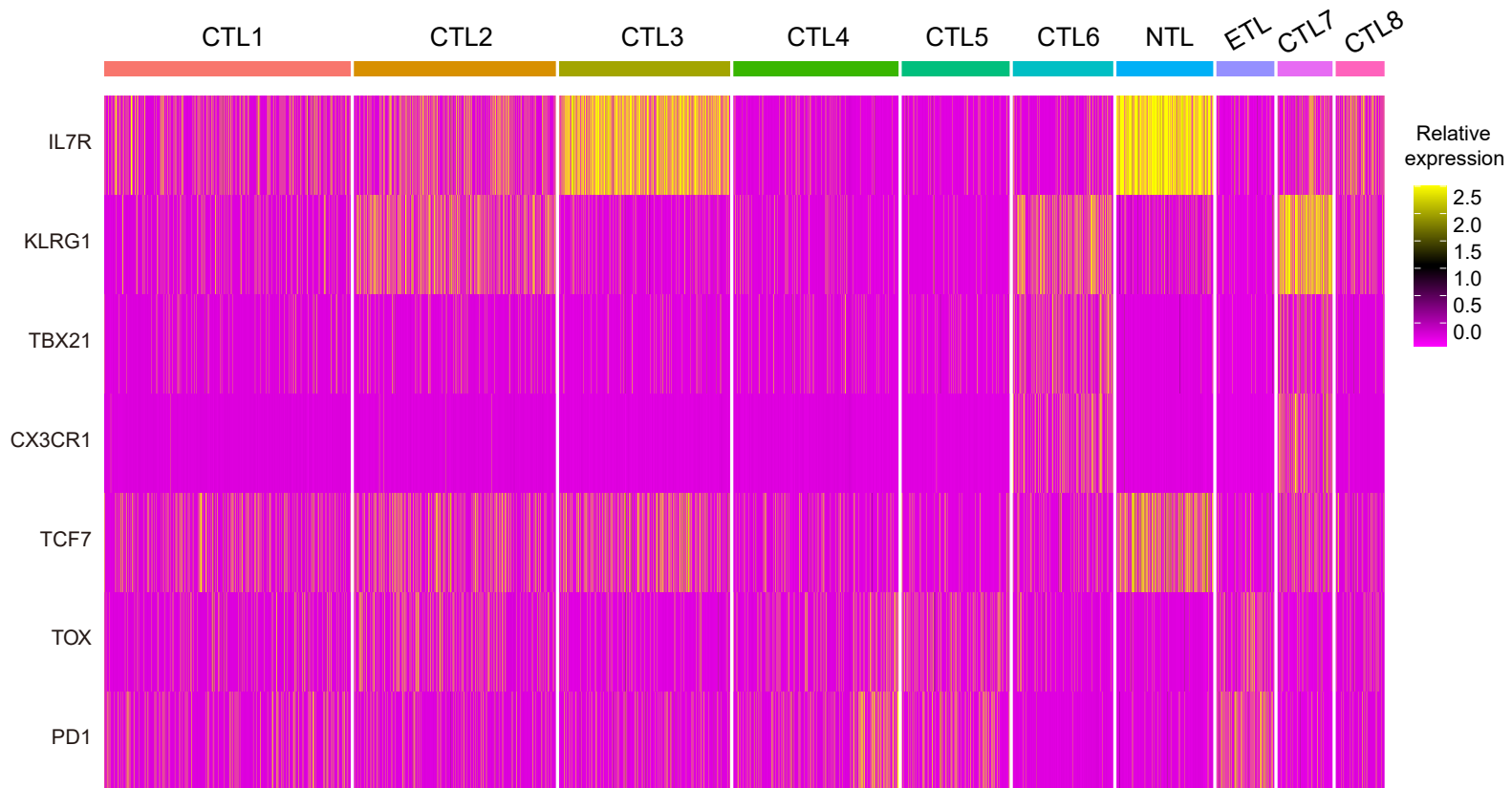

Supplement: Supplementary file 1 [file cancers-14-05183-s001.zip › Figure S3.pdf]

A

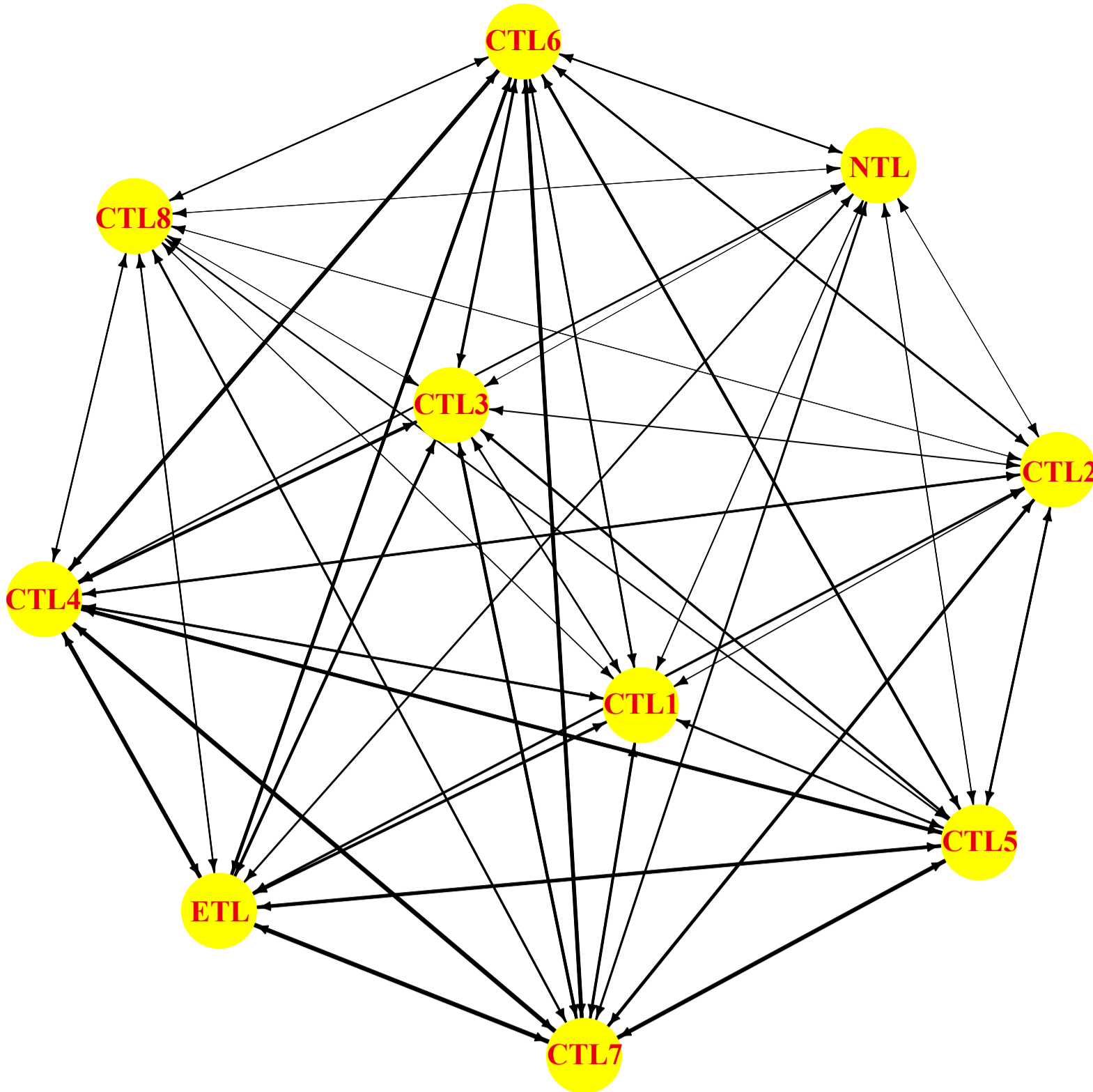

B

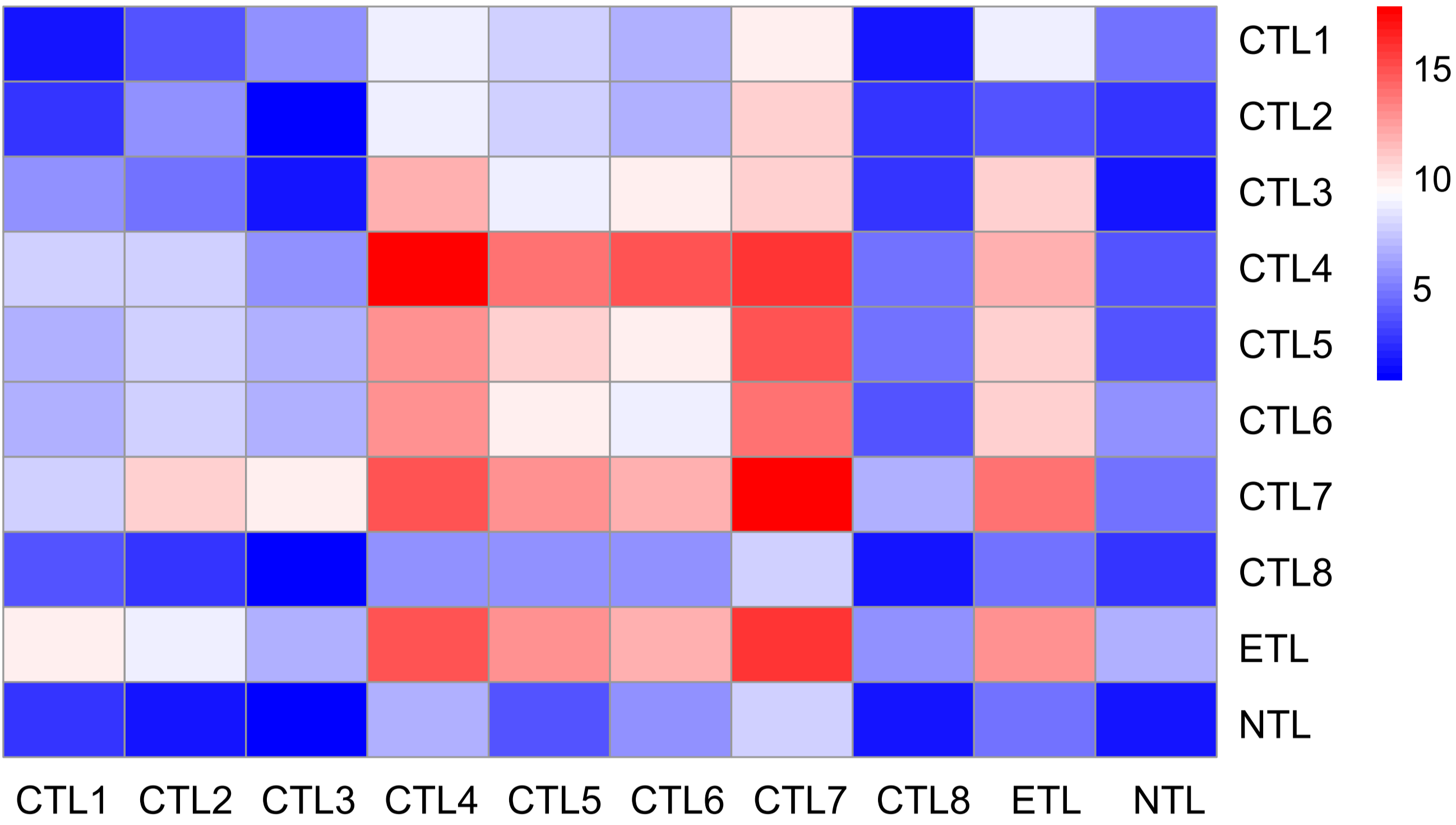

C

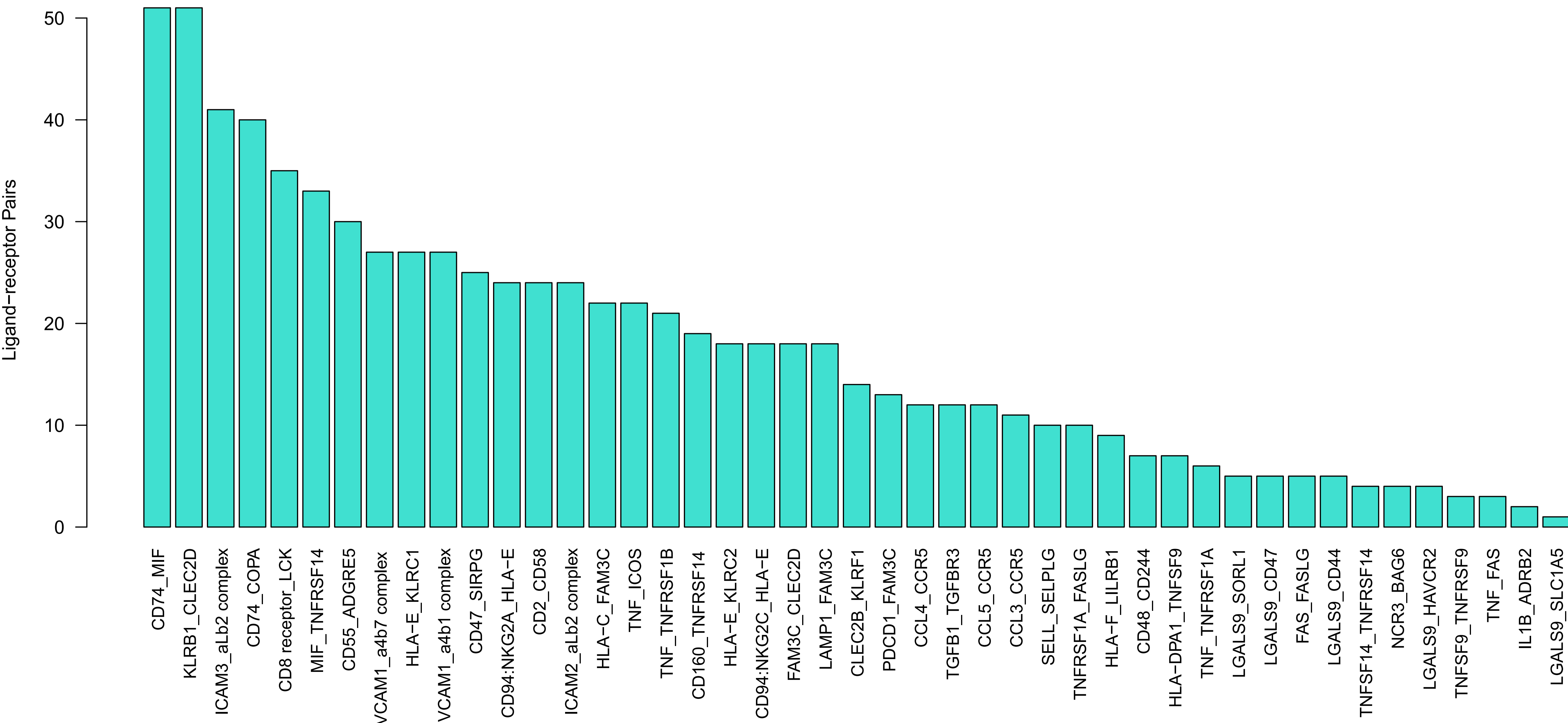

D

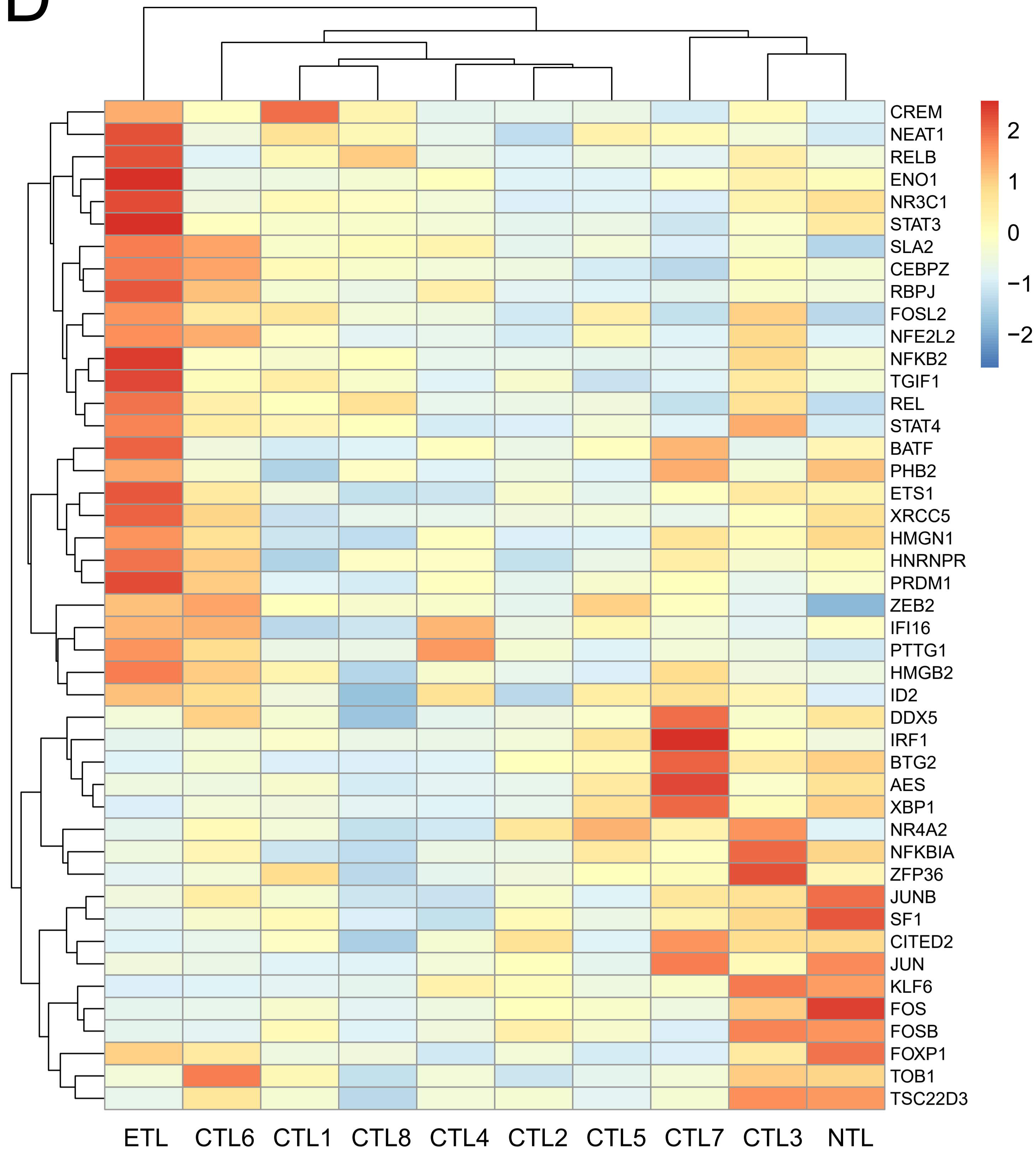

Supplement: Supplementary file 1 [file cancers-14-05183-s001.zip › Figure S4.pdf]

A

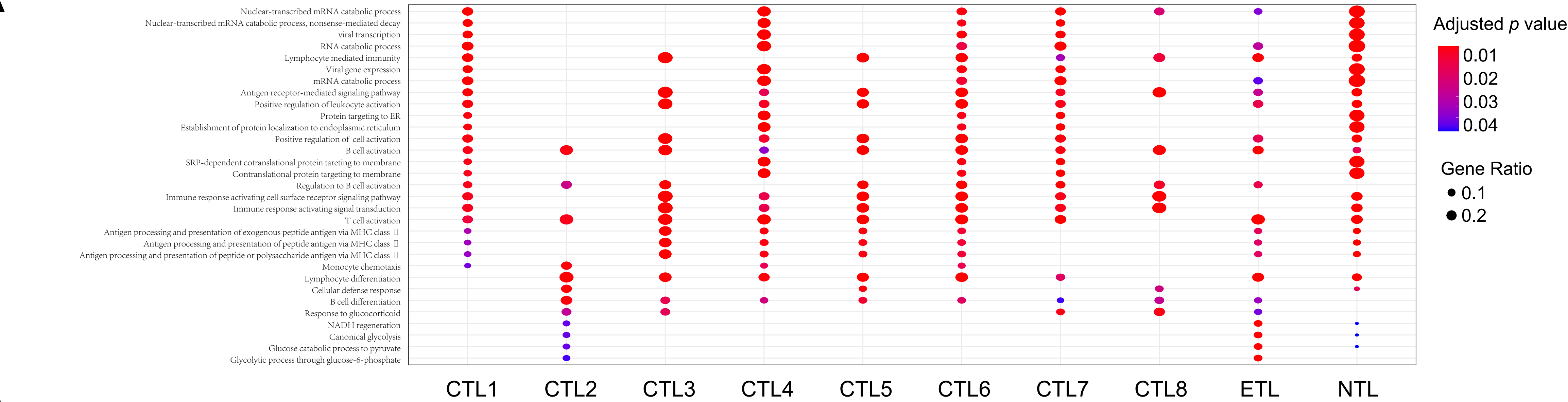

B

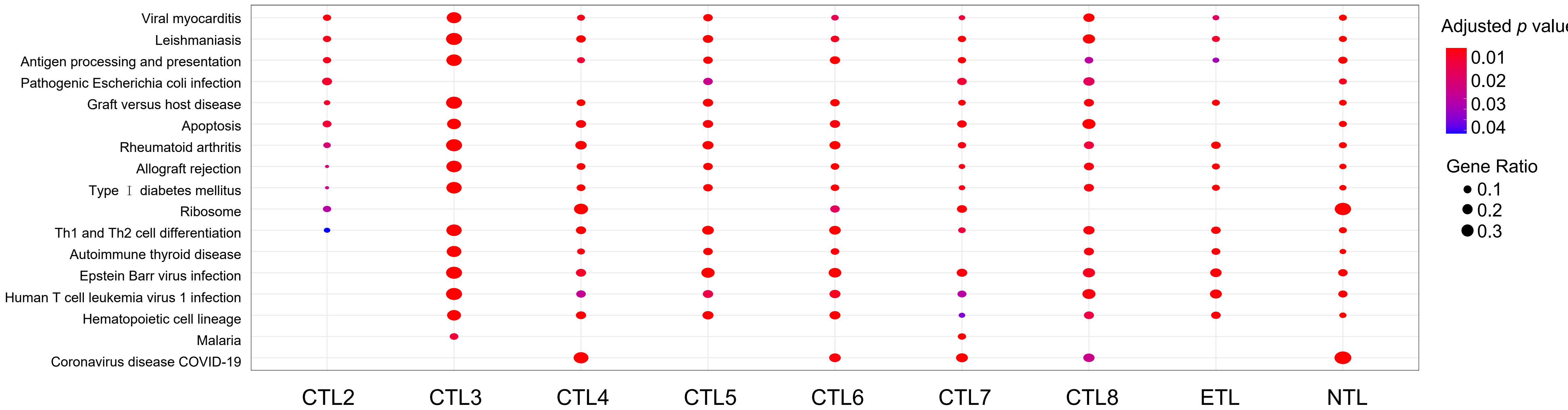

C

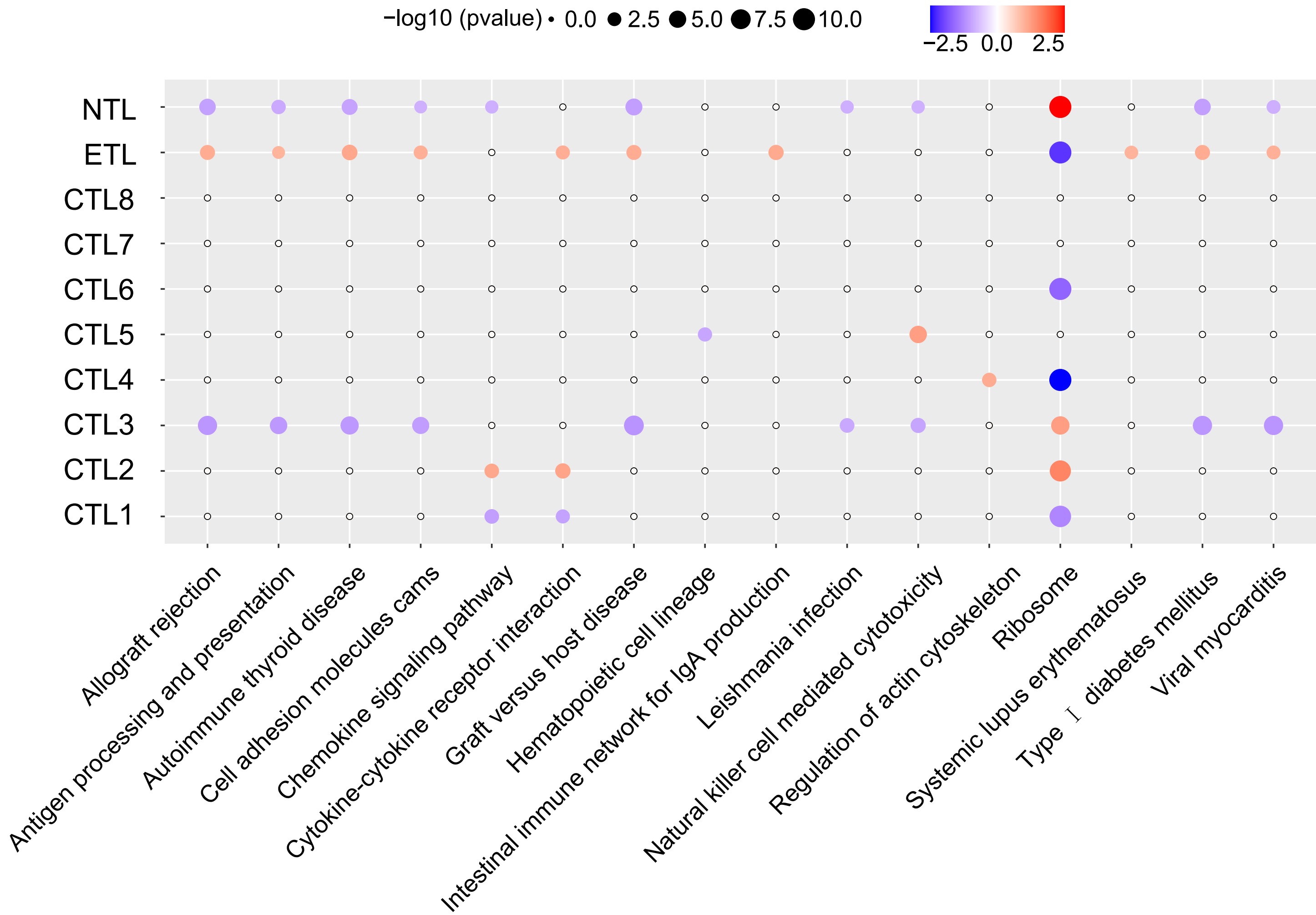

D

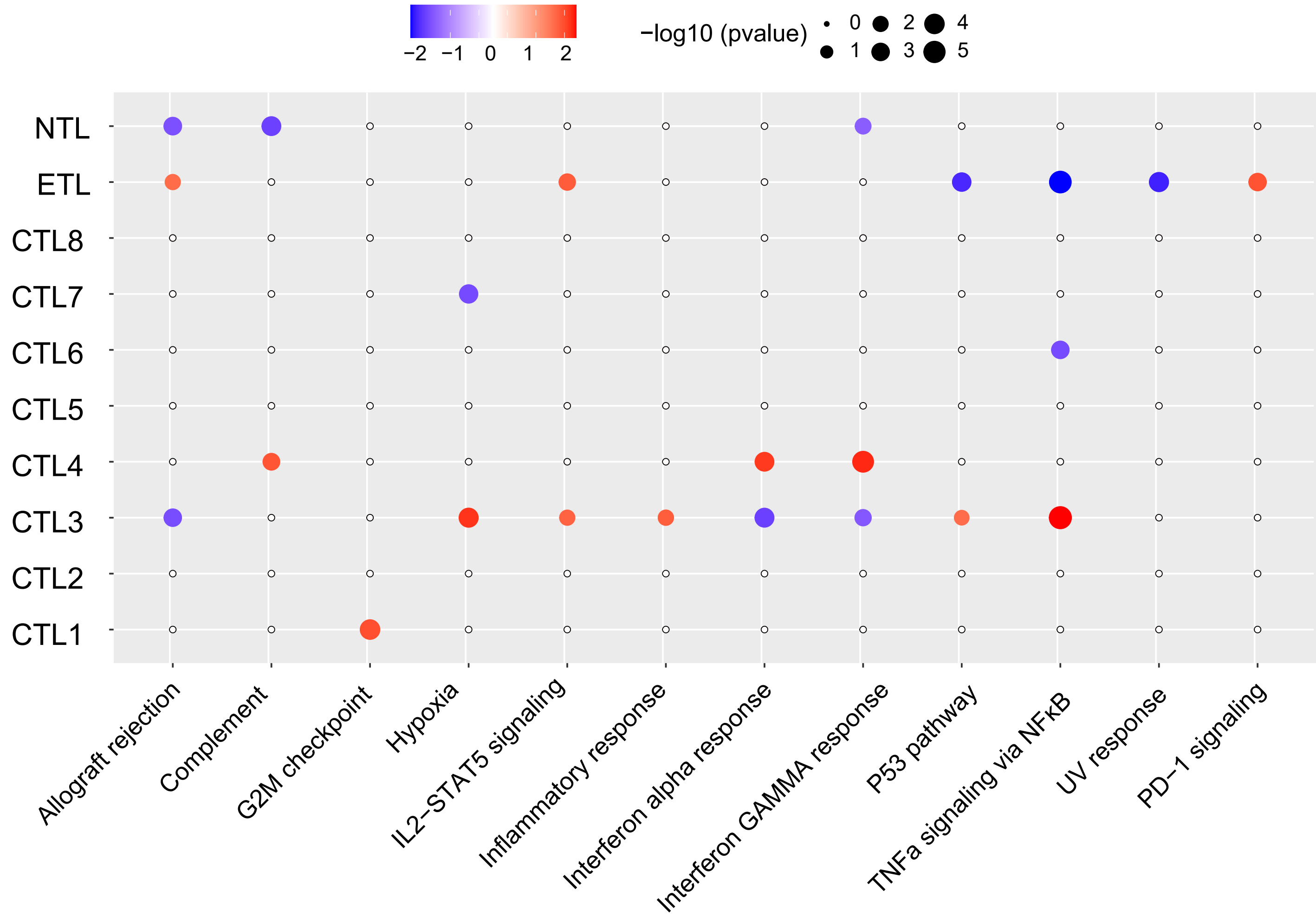

Supplement: Supplementary file 1 [file cancers-14-05183-s001.zip › Figure S5.pdf]

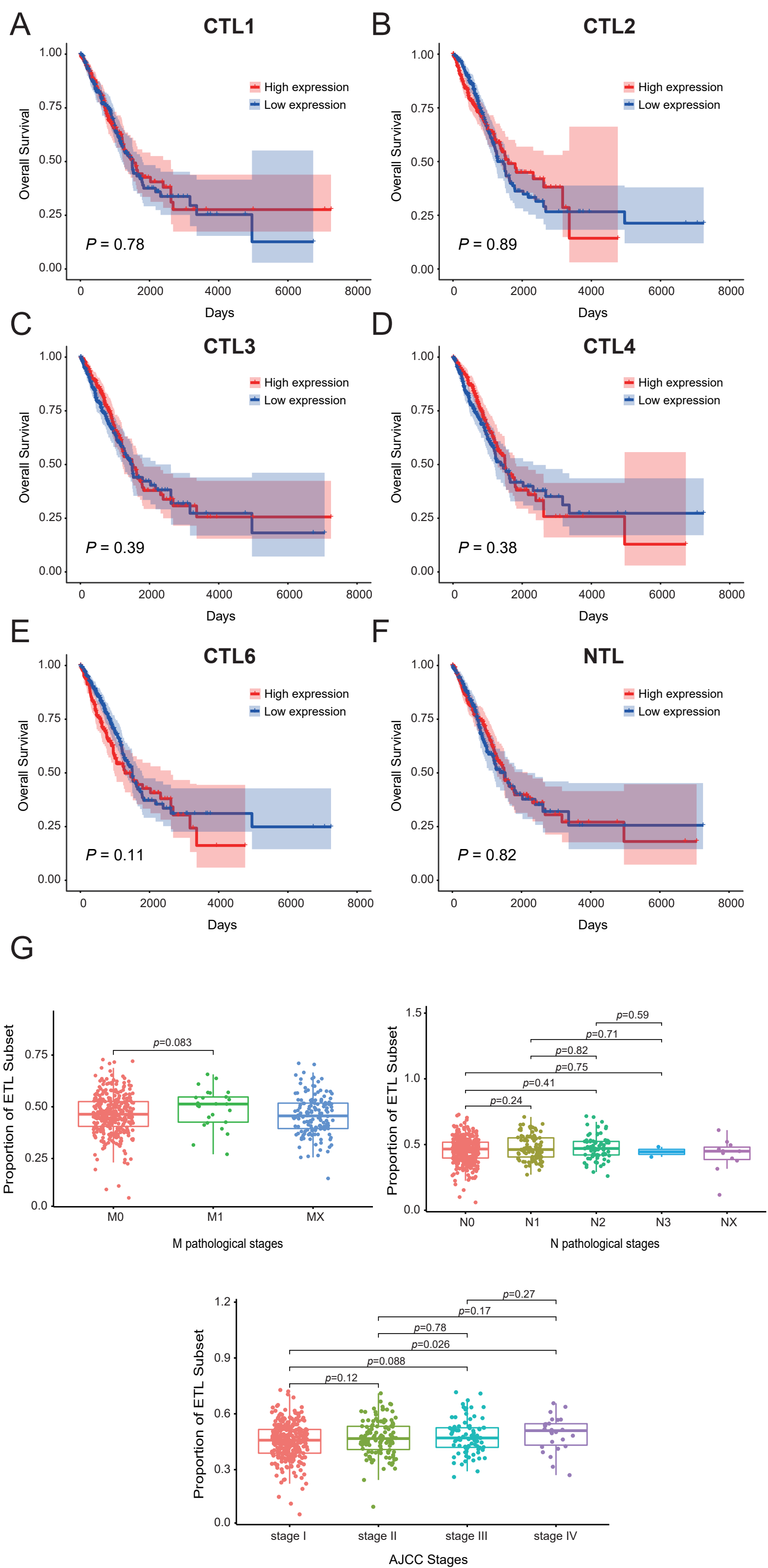

Supplement: Supplementary file 1 [file cancers-14-05183-s001.zip › Figure S6.pdf]

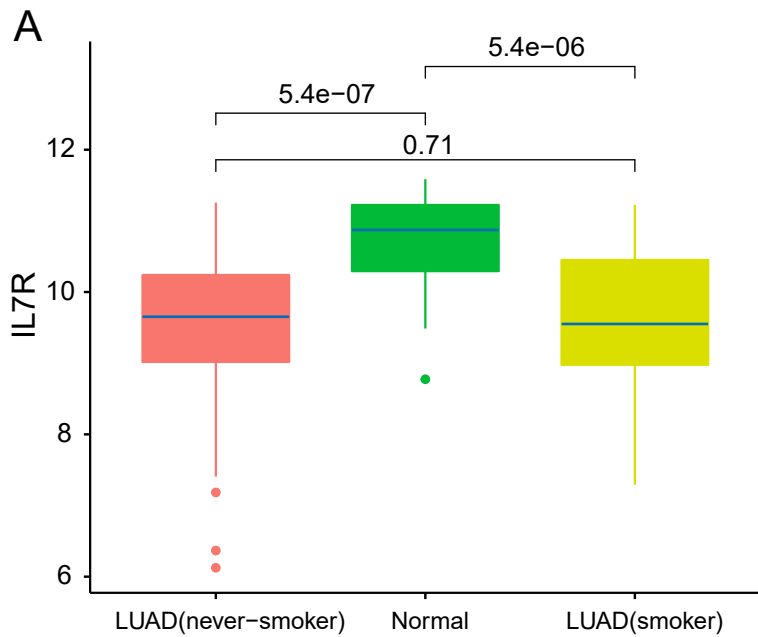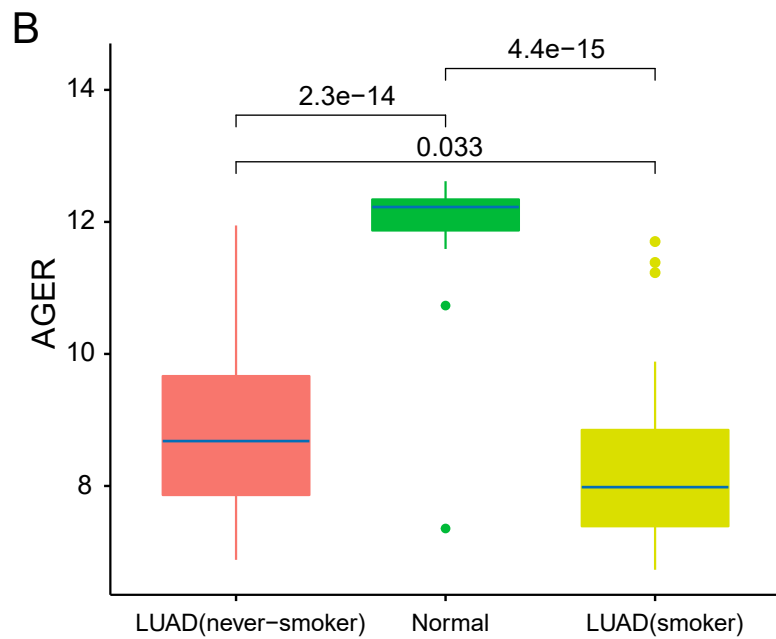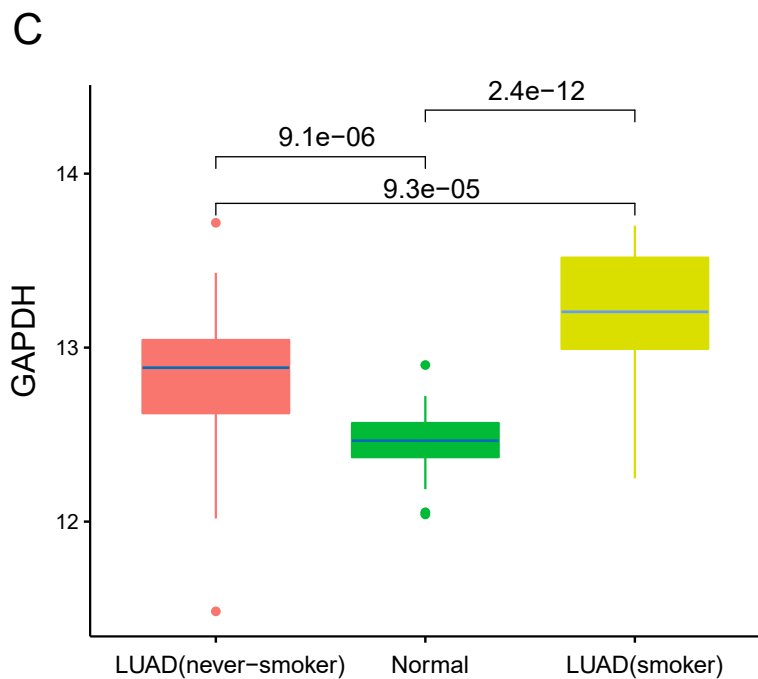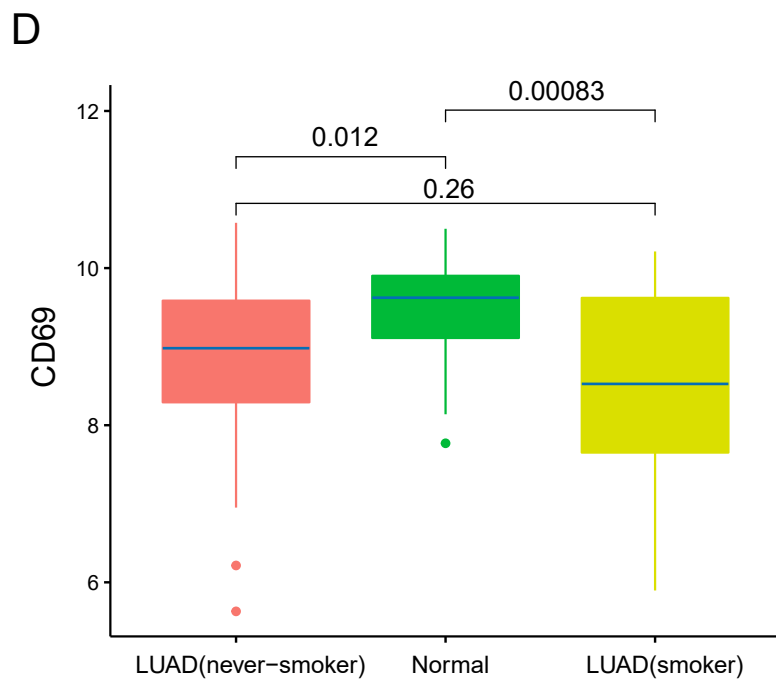

Supplement: Supplementary file 1 [file cancers-14-05183-s001.zip › Figure S7.pdf]

# AGER

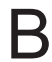

CD69

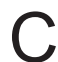

# GAPDH

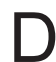

IL7R

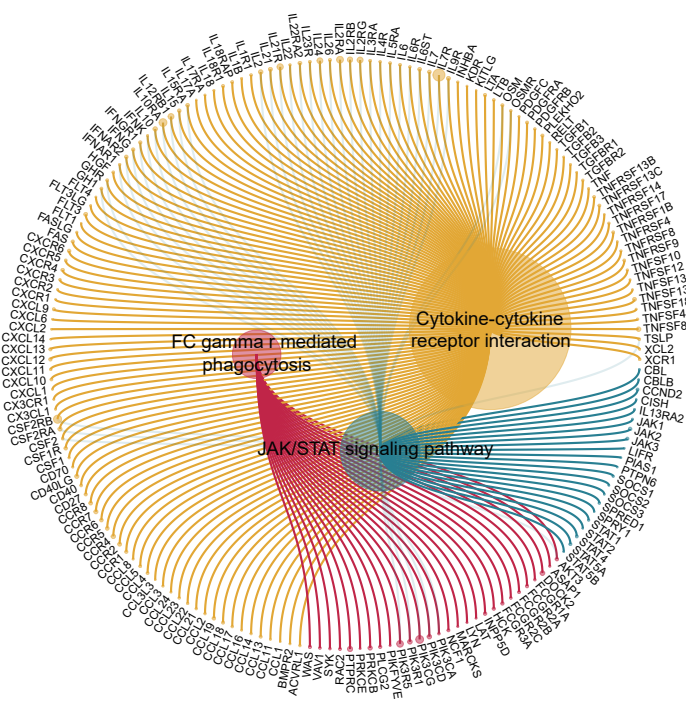

Supplement: Supplementary file 1 [file cancers-14-05183-s001.zip › Figure S8.pdf]
